# West Nile Neuroinvasive Disease Treated With High-Dose Corticosteroids

**DOI:** 10.7759/cureus.31971

**Published:** 2022-11-28

**Authors:** Sarala Kal, Alexander Beland, Mufadda Hasan

**Affiliations:** 1 Internal Medicine, Arrowhead Regional Medical Center, Colton, USA; 2 Emergency Medicine, Arrowhead Regional Medical Center, Colton, USA; 3 Critical Care, Arrowhead Regional Medical Center, Colton, USA

**Keywords:** high-dose corticosteroids, west nile virus infection, west nile, encephalitis, neuroinvasive west nile virus

## Abstract

West Nile neuroinvasive disease, which includes encephalitis, meningitis, or acute flaccid paralysis, is one of the two most common manifestations of West Nile virus (WNV). According to many national agencies, since 1999, WNV has been one of the most common causes of epidemic viral encephalitis in the United States, especially in the state of California, and it will likely remain an important cause of neurological disease for years to come. To date, the mainstay of treatment for West Nile neuroinvasive disease has been supportive care with no data to support the routine use of any agents. Here, we present a unique case of West Nile encephalitis in a 61-year-old male who was successfully treated with a five-day course of high-dose corticosteroids. Although this rapid improvement could be a mere coincidence, it facilitates the need for further trials to determine if high-dose corticosteroids and other drugs may benefit patients in the treatment of West Nile neuroinvasive disease.

## Introduction

Since 2000, West Nile virus (WNV) has been the leading cause of domestically acquired arboviruses. Nearly all human infections of WNV are due to mosquito bites. Birds are the primary amplifying hosts and viruses are maintained in a bird-mosquito-bird cycle [1]. The two most common forms of this avian-borne zoonotic virus are West Nile fever and West Nile neuroinvasive disease. In neuroinvasive disease, WNV infection of neurons resulting in neuronal loss is generally presumed to be the anatomical substrate for the high morbidity and mortality rates. However, on a molecular level, WNV infection also results in a significant upregulation of important proinflammatory molecules that have been reported to promote neuroinflammation and cytotoxicity [2]. Since 2003, there have been more than 7,000 human cases and over 300 deaths from WNV in the state of California, making it the most common and serious vector-borne disease [3].

We present a rare case of West Nile neuroinvasive disease in a 61-year-old male who experienced a prolonged and worsening period of encephalopathy and stupor. The patient had been nonverbal for more than 10 days without any evidence of spontaneous eye movements. Even after treatment with broad-spectrum antibiotics for several days, the patient only experienced marked improvement in mental status on day two of corticosteroid treatment. The vast improvement of encephalopathy occurred by day four as the patient was able to be extubated and follow all commands. The rapid improvement following treatment with an immunosuppressant allows us to hypothesize that a pathogenic post-infectious pro-inflammatory state, rather than neuronal loss, may contribute to morbidity in some cases of WNV neuroinvasive disease [2].

This case report was carried out in accordance with the Institutional Review Boards for Human Research at Arrowhead Regional Medical Center in Colton, CA. The patient gave written informed consent and the protocol was approved by the respective Institutional Review Boards.

## Case presentation

A 61-year-old male working as a security guard presented to the Emergency Department (ED) with the chief complaint of worsening diffuse abdominal pain rated 9/10 for four days prior to presentation. He reported intermittent room spinning sensation, particularly when moving his head or going from a sitting to a standing position. In addition, the patient endorsed dizziness, constipation, nausea, vomiting, urinary hesitancy, fever, and chills. He denied any change in pain with eating or bowel movements, or blood in the stool. He was seen in the ED three days prior and discharged after computed tomography (CT) of the abdomen and pelvis with contrast was unremarkable other than evidence of fatty liver. The patient experienced resolution of pain with one dose each of ondansetron, morphine, and 1 L normal saline. His medical history included diabetes (hemoglobin A1c 6.2) and hypertension for which he was not taking any scheduled home medications. He reported a surgical history of left fourth and fifth toe amputation a year prior, but no family or social history. Physical examination on admission was only significant for mild abdominal pain and weakness (4/5 strength) in the bilateral lower extremities. As the patient showed signs of systemic inflammatory response syndrome with fever, leukocytosis, and tachycardia, with unclear source, he was admitted to the hospital for abdominal pain and intravenous antibiotics, ceftriaxone and vancomycin.

Within two days of admission, the patient’s mental status began to worsen. He complained of urinary retention and showed signs of acute encephalopathy, with Glasgow Coma Scale (GCS) declining from 15 to 10. At that time, there was no evidence of nuchal rigidity on the physical examination. Antibiotics were broadened from ceftriaxone to cefepime and the patient’s head CT without contrast was unremarkable. The next day, the patient was observed to have rigidity in bilateral upper extremities. The patient was found to be alert and oriented to person only with a GCS of 13. Neurology recommended obtaining an electroencephalogram (EEG), lumbar puncture (LP), and brain magnetic resonance image (MRI) without contrast. Metoclopramide was discontinued as a possible cause of rigidity/altered mental status. Repeat head CT without contrast was unremarkable. Empiric coverage for meningitis was begun with ceftriaxone, vancomycin, and acyclovir. Though the patient’s mentation slightly improved the day following broad-spectrum antibiotics, the patient began to rapidly decline the following day and was intubated for airway protection and taken to the Intensive Care Unit (ICU) for worsening encephalopathy. The patient was given lorazepam 2 mg intravenously (IV) for reported tonic-clonic seizure-like activity and started on levetiracetam 500 mg IV twice daily after a third repeat head CT without contrast was unremarkable.

In the ICU, an EEG was done which showed signs of metabolic encephalopathy versus post-ictal state, post-anoxic condition, and autoimmune encephalitis versus paraneoplastic syndrome. A brain MRI without contrast was done that showed no acute intracranial hemorrhage, mass, mass effect, or focal diffusion restriction. LP showed lymphocytic pleocytosis (98%), high glucose (134 mg/dL), and protein (95 mg/dL), along with a high number of white blood cells (38 µL) and red blood cells (106/mm^3^). Further cerebrospinal fluid (CSF) studies for cryptococcus, coccidioidomycosis, herpes simplex virus, measles, VDRL (syphilis), and CSF culture returned negative. We later received positive antibodies for West Nile immunoglobulin (Ig)G (1.66) and West Nile IgM + (7.98). Both the Infectious Disease and Neurology teams suggested a course of steroids (solumedrol 1 g IV daily) for five days based on a single case report published in the state of Mississippi showing marked improvement in clinical status [2]. All antibiotics were subsequently discontinued. For 10 days prior to receiving steroids, the patient was stuporous, unresponsive to commands, and exhibiting the Babinski reflex on the left. Within the first 48 hours of receiving steroids, the patient’s mentation dramatically improved. After being nonverbal for several days, he exhibited spontaneous eye-opening on day two of steroid treatment and was able to fully respond to all commands and was extubated on day four of steroid treatment. He began to speak again within the following two days of treatment completion and was subsequently transferred back to a regular care bed.

## Discussion

While most individuals infected with WNV remain asymptomatic, approximately 1 in 150 develop severe central nervous system (CNS) symptoms [4]. The development of CNS symptoms is associated with increased morbidity and mortality. In particular, mortality for individuals with WNV encephalitis is approximately 12-15% and is likely higher for elderly and immunocompromised individuals [5]. In one large series of 57 patients with neurological sequelae of WNV encephalitis from 2002, 10 patients died (case-fatality rate of 18%), 37 had persistent neurological deficits, the mean length of stay in an ICU was 28 days, only nine recovered fully, and only 13 (23%) were discharged home without extra support [2,6].

There is an increasing body of evidence to suggest that CNS sequelae associated with WNV are not simply a result of direct viral invasion but also exacerbated by an exaggerated host inflammatory response [7,8]. Evidence for post-infectious immune-mediated CNS dysfunction is further bolstered by the fact that over one-third of patients with confirmed WNV CNS disease develop delayed-onset neurological deficits up to one-year post-infection [9]. Currently, there is no Food and Drug Administration-approved treatment for WNV infection, and supportive care is the mainstay of clinical practice.

There have only been a few novel case reports suggesting that corticosteroids may have a neuroprotective effect [2,10-12]. A randomized clinical trial examining the effect of high-dose corticosteroids on patients with a related flavivirus, the Japanese encephalitis virus, demonstrated no improvement in mortality or in return to baseline neurological function [13]. To date, there have been no randomized clinical trials examining the effect of corticosteroids in the treatment of WNV CNS disease. Although the use of corticosteroids in patients with WNV neuroinvasive disease seems counterintuitive, with concern that immunosuppressive effects may promote viremia and worsen outcomes, there is compelling evidence that WNV is rapidly cleared by effective innate and adaptive immune responses within several days of the onset of viremia [2]. Hence, this case report may add to the rising number of clinical case reports showing marked improvement in encephalopathy and neuroinvasive disease associated with WNV.

## Conclusions

WNV with CNS involvement is associated with considerable morbidity and mortality. Currently, supportive care is the mainstay of clinical practice, but there is a growing body of anecdotal evidence, including the case described above, to suggest that corticosteroids have a beneficial neuroprotective effect in select populations. This highlights the need for further randomized control trials examining the treatment with steroids among patients with confirmed WNV CNS disease.
